# Successful Surgical Intervention for External Snapping Hip Syndrome in a Middle‐Aged Woman

**DOI:** 10.1155/cro/1944562

**Published:** 2026-04-21

**Authors:** Mehdi Motififard, Amirarsalan Armanfar, Armin Adibi

**Affiliations:** ^1^ Department of Orthopedic Surgery, Isfahan University of Medical Sciences, Isfahan, Iran, mui.ac.ir; ^2^ Neuroscience Research Center, Isfahan University of Medical Sciences, Isfahan, Iran, mui.ac.ir

**Keywords:** case report, external snapping hip, iliotibial band syndrome

## Abstract

**Background:**

Snapping hip syndrome is a condition characterized by a palpable or audible snap in the hip, particularly during movement. It can be asymptomatic and is categorized into external (lateral) and internal (medial) types. External snapping hip syndrome (ESHS), often due to the iliotibial band (ITB) flipping over the greater trochanter, is more common and usually responds to conservative treatments.

**Case Presentation:**

We present a case of a 44‐year‐old woman who experienced a sensation of hip dislocation and lateral pain in her right hip in the last 3 years which worsened over time. Her symptoms, including pain triggered by hip flexion and external rotation, were not alleviated by different conservative treatments. Imaging studies, including ultrasound and MRI, did not reveal a thickened ITB or bursa abnormalities. Subsequently, the patient underwent ITB release procedure. A longitudinal incision and perpendicular cuts were made on the ITB in the lateral decubitus position, and the trochanteric bursa was resected. Postoperatively, the patient was allowed weight‐bearing with crutches for 1–2 weeks. The patient improved significantly and returned to normal activities within eight weeks.

**Conclusion:**

This case highlights an atypical presentation of ESHS in a patient outside the typical demographic. A clinical diagnosis was made after unrevealing imaging and ruling out common causes of lateral hip pain. After failed conservative management, surgical treatment resulted in complete symptom resolution and no early complications. These findings support considering ESHS in patients with lateral hip pain across age and activity levels and demonstrate the effectiveness of operative management when conservative treatment fails.

## 1. Introduction

Coxa saltans, or snapping hip syndrome, is a condition where a patient experiences or hears a snapping sound or sensation in the hip, particularly during physical activity or lower extremity movements [1]. This condition is usually asymptomatic, often bilateral, and affects 5%–10% of the general population [2, 3]. Snapping hip syndrome is categorized into two types: external (lateral) and internal (medial) [4, 5]. External snapping hip syndrome is more common [6] and is caused by the iliotibial band (ITB) flipping over the greater trochanter, often due to a tight ITB [7].

Treatment typically involves conservative measures [8], with surgery recommended only if conservative approaches are unsuccessful. Herein, we present a case of snapping hip syndrome that did not improve with conservative treatment but showed complete recovery following surgical intervention.

## 2. Case Report

A 44‐year‐old woman presented to the orthopedics clinic with complaints of a sensation of hip dislocation and lateral pain in her right hip. She also reported difficulties with climbing stairs, running, and kneeling. Her symptoms began 3 years ago and had progressively worsened and become more frequent. The pain was triggered by hip flexion, external rotation, and prolonged walking. She denied any traumatic events, congenital hip abnormalities, history of falls, or previous hip issues. Her medical and family histories were unremarkable, and she reported no substance abuse. Physical examination revealed tenderness in the trochanteric region. Hip joint range of motion was normal. Snapping was assessed by extending the flexed hip with the patient in the lateral decubitus position. The snap occurred at approximately 45° of flexion, was audible and slightly palpable in the lateral groin area. The snap reached its maximum level in 90° of flexion. The patient showed no obvious deformity or limp, and the Trendelenburg sign was negative. Radiographs of the hip showed no significant osseous or articular abnormalities.

Standard anteroposterior pelvis radiograph demonstrated normal femoroacetabular alignment with preserved joint spaces. There was no evidence of cam or pincer morphology, hip dysplasia, degenerative changes, or focal osseous abnormalities around the greater trochanter. The cortical contour of the greater trochanter was smooth, with no signs of enthesophytes, calcific tendinopathy, or remodeling.

Magnetic resonance imaging (MRI) of the right hip, performed with T1‐ and T2‐weighted fat‐suppressed sequences, showed intact gluteus medius and minimus tendons without tendinopathy, partial tears, or muscle atrophy. The tensor fasciae latae and gluteus maximus displayed normal morphology and signal characteristics. The ITB appeared normal in thickness, and there was no evidence of trochanteric bursitis, soft‐tissue edema, or fluid accumulation. Articular cartilage and the acetabular labrum were unremarkable, and no intra‐articular pathology was identified. Ultrasound examination of the lateral hip revealed a normal‐appearing ITB without thickening, hypoechoic changes, or fascial irregularity. The gluteus medius and minimus tendon insertions were intact, and no enlargement of the trochanteric bursa or peri‐trochanteric fluid collection was observed.

Conservative treatments, including activity modification, anti‐inflammatory medication, and physical therapy program, were initiated when the patient first visited the clinic in October 2023. The patient completed 6 months of formal physical therapy; however, these measures did not provide lasting relief for the patient′s symptoms. The patient subsequently underwent ITB release.

**Figure 2:**
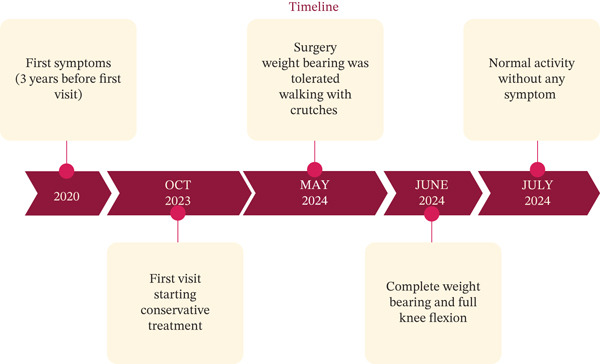
Schematic timeline of the presented case.

Under general anesthesia, the patient was positioned in the lateral decubitus position, with posterior and anterior supports stabilizing the pelvis. The area was prepped and draped in a sterile manner. The greater trochanter was marked, and an incision was made extending from approximately 4 cm above to 10 cm below its tip. A longitudinal 10‐cm incision and three 3‐cm perpendicular incisions were made in the ITB (Figure 1). After releasing the ITB, the trochanteric bursa was identified and resected. Snapping was tested at various stages during the procedure to ensure adequate release. A drain was placed beneath the fascia, and the surgical incision was sutured with nylon. The patient was discharged the day after surgery. Postoperatively, weight‐bearing was allowed as tolerated, with crutches used for comfort for about 1–2 weeks. The postoperative course was uneventful, with no complications. The patient was neurovascularly intact, and the wound was healing well. She began walking with crutches immediately after surgery and progressed gradually. Two weeks postsurgery, she could bear weight and flex the knee fully without symptoms. She returned to normal activities within 8 weeks and, at the 12‐month follow‐up, reported no hip snapping or pain (Figure 2). This case report has been prepared in accordance with the SCARE guidelines [9].

Figure 1(a) After marking greater trochanter, an incision was made, and the iliotibial band was exposed. (b) Subsequently, a longitudinal 10‐cm incision and three 3‐cm perpendicular incisions were made in the iliotibial band.

**Figure:**
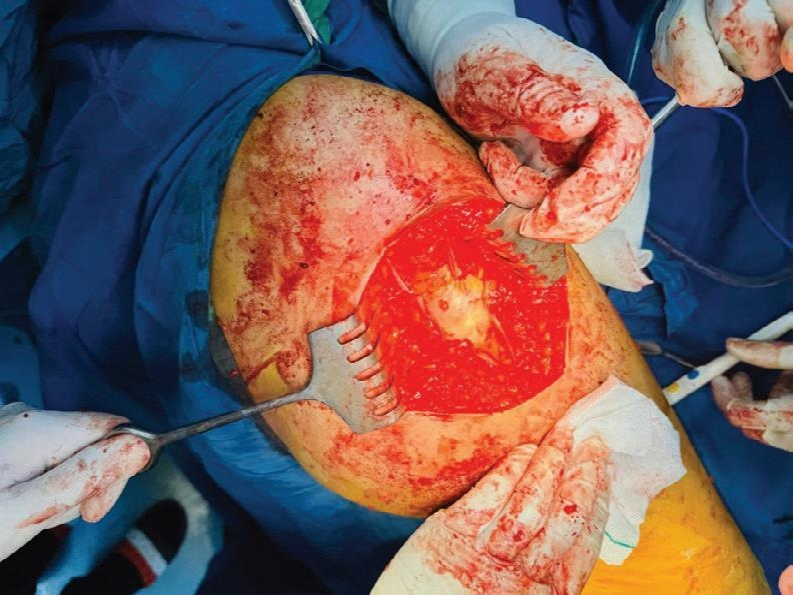
(a)

**Figure:**
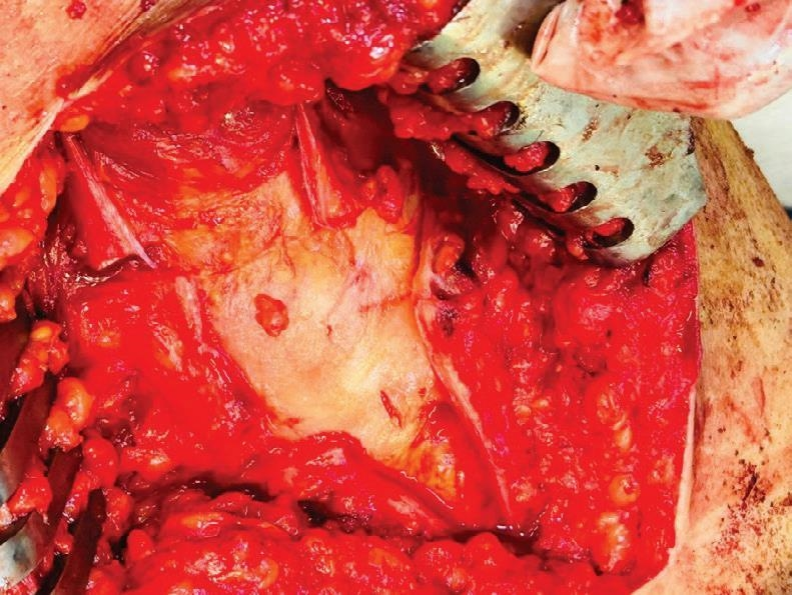
(b)

## 3. Discussion

We present a confirmed case of snapping hip syndrome, which initially manifested as a sensation of dislocation and was neglected for several years. Conservative treatment provided only modest improvement. Following surgical intervention, the patient experienced significant improvement and returned to a normal lifestyle within two months.

External snapping hip syndrome is characterized by a palpable or audible snap on the lateral side of the hip during movement, sometimes accompanied by pain [3]. It is the most common type of snapping hip syndrome, particularly among ballet dancers, runners, and soccer players [10]. Although often asymptomatic, it can be associated with discomfort [8]. This condition is primarily caused by abnormal anterior movement of the ITB over the greater trochanter during hip flexion and external rotation. Less commonly, it results from the gluteus maximus muscle moving abnormally. In most cases, the snapping is related to a thickened ITB, although it can rarely be due to a fibrotic myotendinous junction of the gluteus maximus [11, 12]. Typically, external snapping hip syndrome is not linked to specific occupational or athletic activities or traumatic events, though it is more prevalent in individuals engaged in activities that involve extreme hip motion [13–15]. When painful, snapping hip syndrome is often associated with tendinosis or bursitis due to chronic irritation from friction [16]. Patients commonly experience dull hip pain with an audible snap during hip abduction and external rotation [3].

The diagnosis of external snapping hip syndrome is primarily clinical and is considered a diagnosis of exclusion. Therefore, alternative sources of lateral hip pain, including intra‐articular hip pathology, lumbosacral spine disorders, and neurological causes, must be ruled out. In our patient, the absence of low back pain, radicular symptoms, sensory changes, or motor weakness, along with a normal neurological examination and unremarkable hip imaging, supported the diagnosis of isolated external snapping hip syndrome.

The ITB originates from the tensor fasciae latae and gluteus maximus muscles, running down the lateral thigh and inserting on the tibial Gerdy′s tubercle. It also attaches to the femur and patella. The ITB stabilizes the hip during movement and can become tight with muscle contractions. External snapping hip syndrome occurs when the ITB or gluteus maximus rubs over the greater trochanter, creating a snapping sound, particularly if the ITB is thickened. This snapping can happen during hip flexion or extension [8, 17].

Most cases of external snapping hip resolve with conservative treatment, and surgery is recommended only for cases that do not respond to these measures [18]. Conservative treatment typically includes rest, stretching, anti‐inflammatory medications, corticosteroid injections into the trochanteric bursa, activity modification, and exercises to strengthen the surrounding muscles. Additional therapies may include physical therapy, laser therapy, and extracorporeal shockwave therapy (ESWT) [6, 19–21]. Conservative management should be pursued for at least 6 months before considering surgical options [22].

The goal of surgical treatment for external snapping hip syndrome is to release the contracted ITB to eliminate the snapping [8]. Various surgical techniques, both open and endoscopic, have been shown to yield good outcomes [8]. Although surgery can be an effective option, it may lead to complications including hematoma formation, wound issues, asymmetry of the buttocks and pelvis, Trendelenburg gait, recurrent infections, persistent hip pain, snapping sensations, muscle weakness, tendon injury, and sensory nerve injuries [23, 24–28].

We present a case of external snapping hip syndrome that did not improve with conservative management but achieved complete relief from pain and snapping following surgery.

In a case report by Chang et al., a 34‐year‐old man experienced a snapping sensation in his right hip while running, which began after playing soccer. Similar to our case, static ultrasound did not reveal a thickened ITB. Although the patient′s pain gradually improved, the snapping persisted despite conservative treatment. This highlights that normal imaging studies may not definitively rule out snapping hip syndrome in such cases [12]. Kunac et al. reported two cases of external snapping hip syndrome in 14‐ and 15‐year‐old adolescents, where conservative treatment was ineffective. Both patients underwent endoscopic ITB release and greater trochanteric bursectomy, resulting in complete resolution of symptoms with no surgical complications, and neither patient experienced snapping or pain during a 24‐month follow‐up. Similarly, in our case, conservative treatment failed, but surgical intervention led to significant improvement [29]. Battaglia et al. described a 34‐year‐old woman with an audible click in the lateral left hip for several years. Dynamic ultrasound revealed an abnormal snap associated with anterior dislocation of the gluteus maximus muscle over the greater trochanter. MRI showed a dysmorphic sickle‐shaped myotendinous junction of the gluteus maximus with muscle fatty degeneration. The patient was diagnosed with external snapping hip syndrome secondary to this dysmorphism and improved progressively with nonsteroidal anti‐inflammatory drugs and stretching exercises [3].

In our case, although external snapping hip syndrome is typical of young adults and athletes and often bilateral [30], our patient was a middle‐aged woman with no history of athletic activity, presenting with right‐sided snapping. Despite conservative treatments, including activity modification, anti‐inflammatory medications, and physical therapy, there was no improvement. However, the patient showed remarkable improvement following surgery, with a significant reduction in symptoms.

In conclusion, external snapping hip syndrome should be considered in patients with lateral hip pain regardless of age or athletic history. Normal imaging studies may not definitively rule out the condition, emphasizing the need for a thorough clinical evaluation.

## Author Contributions

Mehdi Motififard and Amirarsalan Armanfar designed the study. Amirarsalan Armanfar supervised the study. Armin Adibi drafted the text. All authors contributed to the manuscript.

## Funding

No funding was received for this manuscript.

## Disclosure

No figures were taken from any journals, websites, and other sources. All authors approved the submitted version.

## Ethics Statement

Written informed consent was obtained from the patient for the publication of this case report. The participant has consented to the submission of the case report to the journal. A copy of the written consent is available for review by the editor in chief of this journal on request. The study was ethically approved by the Ethics Committee of Isfahan University of Medical Sciences, Isfahan, Iran (Ethics Code: IR.ARI.MUI.REC.1403.249).

## Conflicts of Interest

The authors declare no conflicts of interest.

## Data Availability

The data are available from the corresponding authors (Amirarsalan Armanfar) upon reasonable request.

## References

[1] Snapping B. E. , Hip Syndrome, Orthopaedic Nursing. (2018) 37, no. 6, 357–360. 30451771 10.1097/NOR.0000000000000499

[2] Via A. G. , Fioruzzi A. , and Randelli F. , Diagnosis and Management of Snapping Hip Syndrome: A Comprehensive Review of Literature, Rheumatology. (2017) 7, no. 4.

[3] Battaglia M. , Guaraldi F. , Monti C. , Vanel D. , and Vannini F. , An Unusual Cause of External Snapping Hip, Journal of Radiology Case Reports. (2011) 5, no. 10, 1–6, 10.3941/jrcr.v5i10.821, 2-s2.0-80054718850, 22470763. PMC3303464 22470763

[4] Pecina M. M. , Overuse Injuries of the Musculoskeletal System, 1993, CRC Press.

[5] Pećina M. , Bojanić I. , and Hašpl M. , The Snapping Hip, HIP International. (1994) 4, no. 3–4, 133–136, 10.1177/1120700094004003-404.

[6] Allen W. C. and Cope R. , Coxa Saltans: The Snapping Hip Revisited, JAAOS-Journal of the American Academy of Orthopaedic Surgeons. (1995) 3, no. 5, 303–308. 10.5435/00124635-199509000-00006 10790668

[7] Thomassen P. J. B. , Basso T. , and Foss O. A. , Endoscopic Treatment of Greater Trochanteric Pain Syndrome-a Case Series of 11 Patients, Journal of Orthopaedic Case Reports. (2019) 9, no. 1, 6–10, 10.13107/jocr.2250-0685.1284, 31245309. PMC6588147 31245309

[8] Randelli F. , Mazzoleni M. G. , Fioruzzi A. , Giai Via A. , Calvisi V. , and Ayeni O. R. , Surgical Interventions for External Snapping Hip Syndrome, Knee Surgery, Sports Traumatology, Arthroscopy. (2021) 29, 2386–2393. 10.1007/s00167-020-06305-w PMC8298335 33064193

[9] Sohrabi C. , Mathew G. , Maria N. , Kerwan A. , Franchi T. , Agha R. A. , and Collaborators , The SCARE 2023 Guideline: Updating Consensus Surgical CAse REport (SCARE) Guidelines, International Journal of Surgery. (2023) 109, no. 5, 1136–1140, 10.1097/JS9.0000000000000373, 37013953. 37013953 PMC10389401

[10] Sammarco G. J. , The Dancer′s Hip, Clinics in Sports Medicine. (1983) 2, no. 3, 485–498, 10.1016/S0278-5919(20)31381-8, 6652698. 6652698

[11] Brignall C. G. , Brown R. M. , and Stainsby G. D. , Fibrosis of the Gluteus Maximus as a Cause of Snapping Hip, Journal of Bone & Joint Surgery. (1993) 75, no. 6, 909–910. 10.2106/00004623-199306000-00012 8314831

[12] Chang K.-S. , Cheng Y.-H. , Wu C.-H. , and Özçakar L. , Dynamic Ultrasound Imaging for the Iliotibial Band/Snapping Hip Syndrome, American Journal of Physical Medicine & Rehabilitation. (2015) 94, no. 6, e55–e56, 10.1097/PHM.0000000000000299, 2-s2.0-84930016832, 25888667. 25888667

[13] Pelsser V. , Cardinal É. , Hobden R. , Aubin B. , and Lafortune M. , Extraarticular Snapping Hip: Sonographic Findings, American Journal of Roentgenology. (2001) 176, no. 1, 67–73, 11133541, 10.2214/ajr.176.1.1760067, 2-s2.0-0035178608. 11133541

[14] Lewis C. L. , Extra-Articular Snapping Hip: A Literature Review, Sports Health. (2010) 2, no. 3, 186–190, 23015936. 23015936 10.1177/1941738109357298 PMC3445103

[15] Wahl C. J. , Warren R. F. , Adler R. S. , Hannafin J. A. , and Hansen B. , Internal Coxa Saltans (Snapping Hip) as a Result of Overtraining: A Report of 3 Cases in Professional Athletes With a Review of Causes and the Role of Ultrasound in Early Diagnosis and Management, American Journal of Sports Medicine. (2004) 32, no. 5, 1302–1309, 15262657. 15262657 10.1177/03363546503258777

[16] Kingzett-Taylor A. , Tirman P. F. , Feller J. , McGann W. , Prieto V. , Wischer T. , Cameron J. A. , Cvitanic O. , and Genant H. K. , Tendinosis and Tears of Gluteus Medius and Minimus Muscles as a Cause of Hip Pain: MR Imaging Findings, AJR. American Journal of Roentgenology. (1999) 173, no. 4, 1123–1126, 10511191. 10511191 10.2214/ajr.173.4.10511191

[17] Sher I. , Umans H. , Downie S. A. , Tobin K. , Arora R. , and Olson T. R. , Proximal Iliotibial Band Syndrome: What Is It and Where Is It?, Skeletal Radiology. (2011) 40, no. 12, 1553–1556, 10.1007/s00256-011-1168-5, 2-s2.0-85027917377, 21499978. 21499978

[18] Ilizaliturri V. M. , Martinez-Escalante F. A. , Chaidez P. A. , and Camacho-Galindo J. , Endoscopic Iliotibial Band Release for External Snapping Hip Syndrome, Arthroscopy. (2006) 22, no. 5, 505–510, 16651159. 16651159 10.1016/j.arthro.2005.12.030

[19] Frizziero A. , Vittadini F. , Pignataro A. , Gasparre G. , Biz C. , Ruggieri P. , and Masiero S. , Conservative Management of Tendinopathies Around Hip, Muscles, Ligaments and Tendons Journal. (2016) 6, no. 3, 281–292, 10.11138/mltj/2016.6.3.281, 2-s2.0-85007188366, 28066732. 28066732 PMC5193517

[20] Yen Y.-M. , Lewis C. L. , and Kim Y.-J. , Understanding and Treating the Snapping Hip, Sports Medicine and Arthroscopy Review. (2015) 23, no. 4, 194–199, 10.1097/JSA.0000000000000095, 2-s2.0-84947055860, 26524554. 26524554 PMC4961351

[21] Zoltan D. J. , Clancy W. G. , and Keene J. S. , A New Operative Approach to Snapping Hip and Refractory Trochanteric Bursitis in athletes, American Journal of Sports Medicine. (1986) 14, no. 3, 201–204, 3752359. 3752359 10.1177/036354658601400304

[22] Pierce T. P. , Kurowicki J. , Issa K. , Festa A. , Scillia A. J. , and McInerney V. K. , External Snapping Hip: A Systematic Review of Outcomes Following Surgical Intervention: External Snapping Hip Systematic Review, Hip International. (2018) 28, no. 5, 468–472, 10.1177/1120700018782667, 2-s2.0-85053196864, 29902932. 29902932

[23] Giai Via R. , Elzeiny A. , Pantè S. , De Vivo S. , Massè A. , and Giachino M. , Can We Encourage the Endoscopic Treatment for External Snapping Hip (ESH)? A Systematic Review of Current Concepts, European Journal of Orthopaedic Surgery & Traumatology. (2024) 34, no. 6, 2835–2844, 10.1007/s00590-024-04030-5, 38874780. 38874780 PMC11377505

[24] Tsatlidou M. , Tzaveas A. P. , Kyriakidis T. , and Iosifidis M. , Endoscopic Iliotibial Band Release Is an Effective Treatment for External Snapping Hip Syndrome: A Case Series, Cureus. (2025) 17, no. 8, e91048, 10.7759/cureus.91048, 41018448. 41018448 PMC12463389

[25] Shrestha A. , Wu P. , Ge H. , and Cheng B. , Clinical Outcomes of Arthroscopic Surgery for External Snapping Hip, Journal of Orthopaedic Surgery and Research. (2017) 12, no. 1, 10.1186/s13018-017-0584-1, 2-s2.0-85020027928, 28577354. PMC5455077 28577354

[26] Potalivo G. and Bugiantella W. , Snapping Hip Syndrome: Systematic Review of Surgical Treatment, Hip International. (2017) 27, no. 2, 111–121, 10.5301/hipint.5000464, 2-s2.0-85016976360. 28222210

[27] Malinowski K. , Mostowy M. , Kim D. W. , Bawor M. , Skowronek P. , Hirschmann M. T. , Pękala P. A. , and LaPrade R. , Gluteal Complex Is Important in External Snapping Hip: Intraoperative Identification of Syndrome Origin and Endoscopic Stepwise Release-a Case Series, International Orthopaedics. (2024) 48, no. 2, 401–408, 10.1007/s00264-023-05961-0, 37668725. 37668725 PMC10799799

[28] Dai Z. , Chen Z. , Liao Y. , Tang Z. , and Cui J. , Comparison of Arthroscopic Versus Open Surgery on External Snapping Hip Caused by Gluteal Muscle Contracture, Hip International. (2018) 28, no. 2, 173–177, 10.1177/1120700017754013, 2-s2.0-85051241435. 29890911

[29] Kunac N. , Tršek D. , Medančić N. , Starčević D. , and Hašpl M. , Endoscopic Treatment of the External Snapping Hip Syndrome: Surgical Technique and Report of Two Cases, Acta Clinica Croatica. (2012) 51, no. 4, 661–666, 23540176. 23540176

[30] Walker P. , Ellis E. , Scofield J. , Kongchum T. , Sherman W. F. , and Kaye A. D. , Snapping Hip Syndrome: A Comprehensive Update, Orthopedic Reviews. (2021) 13, no. 2, 10.52965/001c.25088. PMC8567760 34745476

